# Tiger sharks support the characterization of the world’s largest seagrass ecosystem

**DOI:** 10.1038/s41467-022-33926-1

**Published:** 2022-11-01

**Authors:** Austin J. Gallagher, Jacob W. Brownscombe, Nourah A. Alsudairy, Andrew B. Casagrande, Chuancheng Fu, Lucy Harding, S. David Harris, Neil Hammerschlag, Wells Howe, Antonio Delgado Huertas, Sami Kattan, Andrew S. Kough, Andre Musgrove, Nicholas L. Payne, Adrian Phillips, Brendan D. Shea, Oliver N. Shipley, U. Rashid Sumaila, Mohammad S. Hossain, Carlos M. Duarte

**Affiliations:** 1 Beneath The Waves, PO Box 126, Herndon, VA USA; 2 Department of Biology, Carleton University, 1125 Colonel By Drive, Ottawa, ON Canada; 3 Red Sea Research Center and Computational Biosciences Research Center, King Abdullah University of Science and Technology, Thuwal, Saudi Arabia; 4 ABC4Films, Cinema Science Division, Naples, FL USA; 5 Trinity College Dublin, Dublin 2, Ireland; 6 Rosenstiel School of Marine and Atmospheric Science, University of Miami, Miami, FL 33149 USA; 7 Instituto Andaluz de Ciencias de la Tierra (CSIC‐UGR), Granada, Spain; 8 Daniel P. Haerther Center for Conservation and Research, John G. Shedd Aquarium, 1200S Lake Shore Drive, Chicago, IL USA; 9 Bahamas Dive Guides, Nassau, New Providence Bahamas; 10 Fisheries Economics Research Unit, University of British Columbia, Vancouver, BC Canada; 11 Institute of Oceanography and Environment (INOS), Universiti Malaysia Terengganu (UMT), 21030 Kuala Nerus, Terengganu Malaysia

**Keywords:** Zoology, Ecosystem services

## Abstract

Seagrass conservation is critical for mitigating climate change due to the large stocks of carbon they sequester in the seafloor. However, effective conservation and its potential to provide nature-based solutions to climate change is hindered by major uncertainties regarding seagrass extent and distribution. Here, we describe the characterization of the world’s largest seagrass ecosystem, located in The Bahamas. We integrate existing spatial estimates with an updated empirical remote sensing product and perform extensive ground-truthing of seafloor with 2,542 diver surveys across remote sensing tiles. We also leverage seafloor assessments and movement data obtained from instrument-equipped tiger sharks, which have strong fidelity to seagrass ecosystems, to augment and further validate predictions. We report a consensus area of at least 66,000 km^2^ and up to 92,000 km^2^ of seagrass habitat across The Bahamas Banks. Sediment core analysis of stored organic carbon further confirmed the global relevance of the blue carbon stock in this ecosystem. Data from tiger sharks proved important in supporting mapping and ground-truthing remote sensing estimates. This work provides evidence of major knowledge gaps in the ocean ecosystem, the benefits in partnering with marine animals to address these gaps, and underscores support for rapid protection of oceanic carbon sinks.

## Abstract (web summary)

This study characterizes the world’s largest seagrass ecosystem in The Bahamas by integrating spatial estimates with remote sensing and performing extensive ground-truthing of benthic habitat with 2,542 diver surveys, as well as data obtained from instrument-equipped tiger sharks, which have strong fidelity to seagrass ecosystems.

## Introduction

Seagrass ecosystems play an increasingly recognized role in supporting biological productivity^1^, carbon sequestration^2,3^, ocean biodiversity^4^ and fishery resources^5^. Seagrasses trap and permanently store massive amounts of carbon in the sediment, contributing an estimated 17% of the total organic carbon annually buried in marine sediments^2^. Rapid seagrass losses over previous decades^6,7^ have reduced the sequestering capacity of seagrass ecosystems, while also releasing large amounts of carbon to the atmosphere^8^. Hence, the conservation of seagrass ecosystems is of critical global importance to manage greenhouse gas emissions while safeguarding the many threatened species and seafood resources supported by seagrass habitat^9^.

Conserving seagrass ecosystems requires, at a minimum, reliable knowledge of their distribution and extent. Yet, they remain poorly mapped across many regions, such that current uncertainty surrounding estimates of global seagrass extent ranges 10-fold^10^, from a recently verified global area of 160,387 km^2 ^^11^^,^ to a predicted area, using niche models delineating suitable space, of 1,600,000 km^2 ^^12^^,^. The global area of seagrass is the main driver of uncertainty on their global carbon sequestration capacity and, thus, their value as blue carbon resources^3,10^. This knowledge gap is a major reason why seagrass ecosystems remain underrepresented in marine protected areas^13^, and therefore, highlighting a clear a focus for the UN Decade of Ocean Science.

The main roadblock to improving the estimation of seagrass area stems from the difficulties associated with resolving them from remote sensing products, because of the known optical overlap between seagrass and overlaying phytoplankton and macroalgae^14^, and in lower-latitude areas, backscatter created from carbonate sediments^15^. Dominant seagrass species, such as *Halophila* sp., typically produce a sparse cover, whereby the canopy only protrudes one or several centimeters above the sediment as it is often partially covered by oolitic sand^16^. As a result, small seagrass ecosystems are still being discovered, and it is likely that very large ones remain unmapped. For instance, the largest known seagrass ecosystem occupies 40,000 km^2^ in the lagoon between the Australian mainland and the Great Barrier Reef and is dominated by small *Halophila* meadows. This seagrass ecosystem was only discovered by SCUBA divers and towed cameras in 2009^17^, despite the Great Barrier Reef being a world-renowned national park since 1975 and arguably one of the most intensely studied marine ecosystems in the world.

**Fig. 1:**
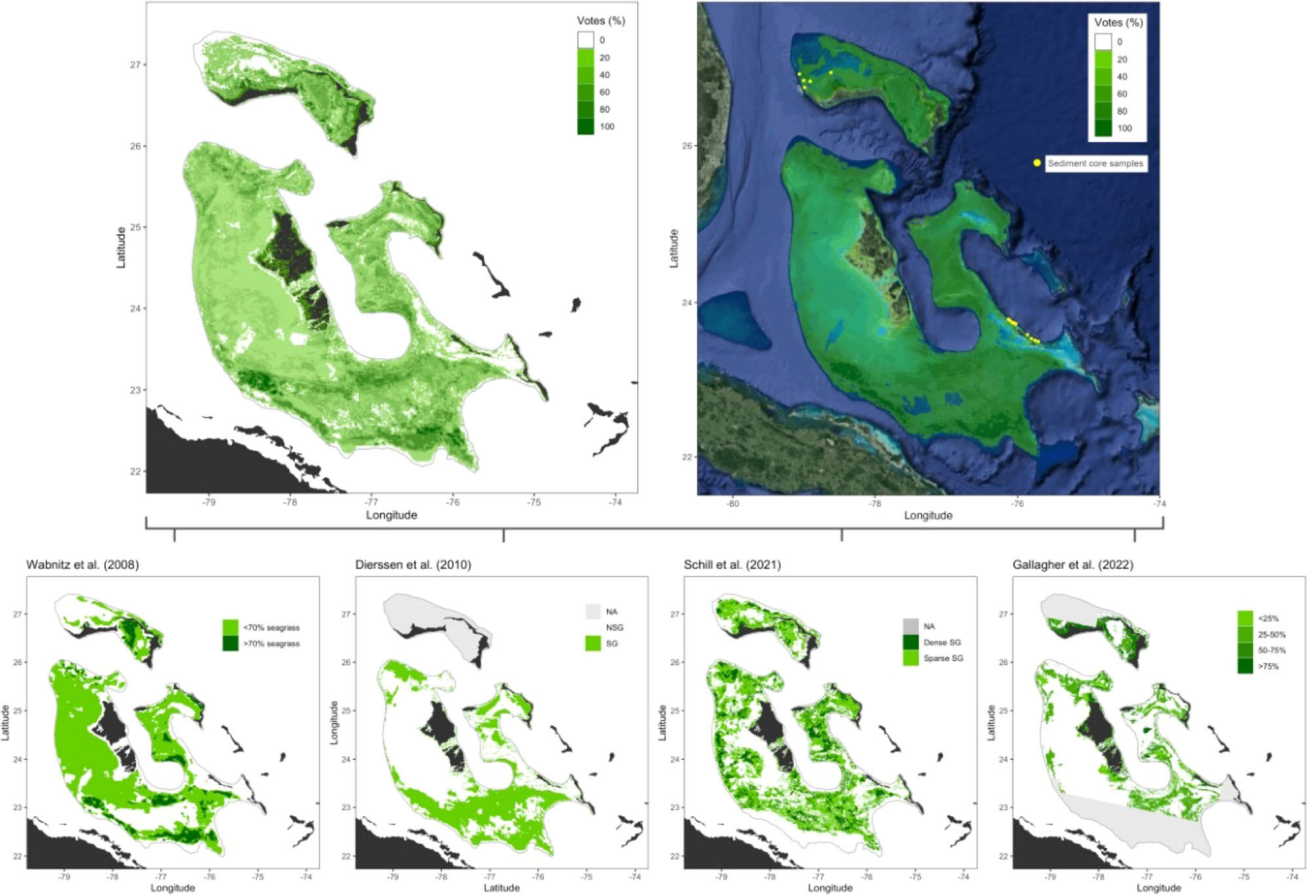
Estimates, based on remote sensing, of seagrass cover on The Bahama Banks. Data from existing assessments^18–20^ and a new assessment derived here (2022; bottom row). Gray regions (NA) indicate areas where no estimates were derived from each remote sensing source (panels in bottom row). Top panels represent aggregated estimates of seagrass coverage from the three sources through an ensemble votes system (see Supplementary Tables 1 and 2 for details) on an open map (left; black indicates land) and satellite map (right). Yellow dots represent empirical sediment cores which were collected and analyzed for quantification of organic carbon stock.

The Bahama Banks are large (>135,000 km^2^), expansive areas defined by widespread carbonate sediments supporting high biodiversity of large, highly mobile consumers (sharks, turtles, dolphins, manatees). They are composed of two separated large banks, the Great Bahama and Little Bahama Banks, with a mean depth <10 m and steep slopes at their boundaries (Fig. 1) and are overlaid by some of the clearest waters in the ocean. The substrate, carbonate sand, warm temperature regime, and ample light reaching to the seafloor are suitable conditions for seagrass, with the suitable plateau area of the banks estimated here at 112,537 km^2^, thereby encompassing most (83%) of the Bahama Banks. Hence, the Bahama Banks may well host the largest seagrass ecosystem in the world. Yet, the current area of seagrass in the Bahama Banks range 30-fold, from a documented area of 2250 km^2 ^^11^, ~8500 km^2^ predicted from a global seagrass niche model^12^, and ~40,000 km^2^ to 65,463 km^2^ predicted from remote sensing^18–20^. These major discrepancies, likely driven by issues related to lacking or limited ground-truthing, are not necessarily surprising, considering the significant logistical challenges and massive financial costs of using human divers to take photos over large marine areas, resulting in existing ground-truthing limited to a specific section of the Bahamas Banks comprising less than 5% of the total area^19^. However, the resulting uncertainty in spatial estimates significantly limits our understanding of the global distribution and conservation needs of this potentially globally significant blue carbon ecosystem.

Recent innovations in the development and application of animal-borne camera tags offer a unique opportunity for biologically relevant ground truthing and classification of habitat that extends well beyond human capacity^21,22^. This is because highly mobile animals can move great distances over short periods of time and are not limited by human-based logistical constraints (e.g., boat and bottom time and range, personnel, sea states, survey depth). Indeed, green turtles have been shown to guide the discovery of seagrass ecosystems^22^. Likewise, the suite of highly mobile fishes utilizing shallow seagrass habitats around the world could serve as critical allies in guiding the discovery of novel seagrass ecosystems, and, more importantly, aiding in ground-truthing established approaches for estimating seagrass spatial extent, such as remote sensing images obtained from Earth-orbiting satellites. Tiger sharks are wide-ranging, apex predators that exhibit high consistent associations with seagrass ecosystems across the globe. All life stages of this species are found widely throughout the expansive marine ecosystem of The Bahamas^23^, with large adults migrating long distances over the banks and forming concentrated aggregations at seasonal hotspots. Previous monitoring efforts of tiger sharks in The Bahamas demonstrated strong habitat selection for seagrass habitats^23^. Therefore, tiger sharks may serve as useful research tools for guiding and refining the mapping of seagrass throughout its range.

**Table 1:** Sediment C_org_ stocks in Bahamas seagrass ecosystems

Core depth (cm)	No. cores	Mean ± 95% CI (Mg C_org_ ha^−1^)	SD (Mg C_org_ ha^−1^)	SE (Mg C_org_ ha^−1^)	Area (km^2^)	Corg stock (Tg)
0–30	21	20.5 ± 2.1	4.9	1.1	66,990	0.14–0.19
0–100	21	68.5 ± 6.9	16.2	3.5	−92,524	0.46–0.63

Source data are provided as a Source Data file.

Here we utilized instrument-equipped tiger sharks (*Galeocerdo cuvier*) to collect seafloor imagery and validate the characterization of expansive seagrass ecosystems on the Bahama Banks. Combined with additional human observations, we expand existing ground-truthing efforts for seagrass mapping by 10-fold to generate a relevant estimate of seagrass area from Landsat 8 images. To buffer against the limitations of individual remote sensing estimates and methods, we integrated our empirical estimate with previous data on seagrass coverage on the banks to yield a composite, consensus area. Using this information, we demonstrate that The Bahama Banks is the world’s largest seagrass ecosystem, with an estimated area of at least 66,900 km^2^ (Fig. 1). This extends the documented seagrass area by 41% relative to current estimates, reaching 227,287 km^2^, underscoring the importance of this region as a globally relevant blue carbon ecosystem (Table 1).

## Results

**Fig. 2:**
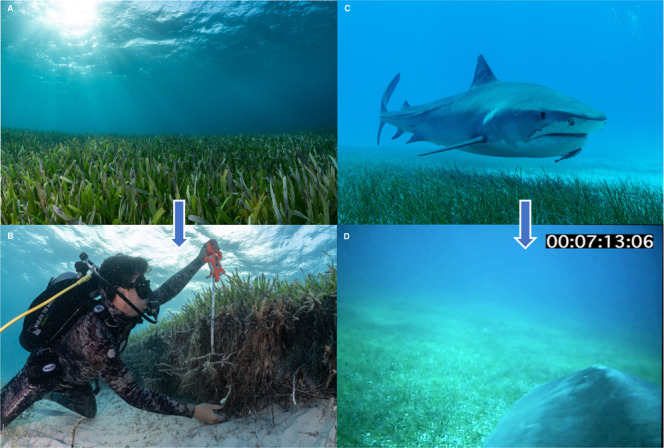
Seagrass meadows on The Bahamas Banks, as surveyed by humans and tiger sharks. **A**, **B** Dense meadow of *Thalassia testudinum* photographed in the southern Great Bahama Bank showing natural erosion scarp exposing the ~1 meter thick root rhizosphere (both images credit: Cristina Mittermeier). **C** Mature tiger shark swimming over *Syringodium filiforme* on the Little Bahama Bank (image credit: Austin Gallagher). **D** POV from camera-mounted tiger shark swimming over dense area of *Thalassia testudinum* on the northern Great Bahama Bank (image credit: Tiger Shark from study).

We conducted 2542 individual surveys assessing the presence of seagrass across the Bahama Banks, where 42% contained dense seagrass meadows, 36% sparse seagrass cover, and 22% did not contain seagrass (Supplementary Fig. 1). Dense meadows were dominated by *Thalassia testudinum*, the largest and climax species, with *Halodule wrightii* and *Syringodium filiforme* in the understory, while sparse meadows consisted of *Halophila decipiens* and *Halodule wrightii* scattered around carbonate sediment (Fig. 2). Empirical explorations of the organic carbon stock of these seagrass ecosystems, based on extensive sediment coring on both Bahama Banks ecosystems containing monospecific meadows of either *T. testudinum* or *S. filiforme* (15 sites comprising 21 sediment cores), delivered the following average total organic carbon contents: *T. testudinum* (*n* = 17 cores/145 sliced samples) *=* 0.006 ± 0.002 g C_org_ cm^−3^ (mean ± SD), range *=* 0.001–0.014 cm^−3^; *S. filiforme* (*n* = 4 cores/32 sliced samples) *=* 0.008 ± 0.002 g C_org_ cm^−3^ (mean ± SD), range *=* 0.005–0.014 g C_org_ cm^−3^; both species combined (*n* = 21 cores/177 sliced samples) *=* 0.007 ± 0.002 g C_org_ cm^−3^ (mean ± SD), range *=* 0.001–0.014 g C_org_ cm^−3^.

**Fig. 3:**
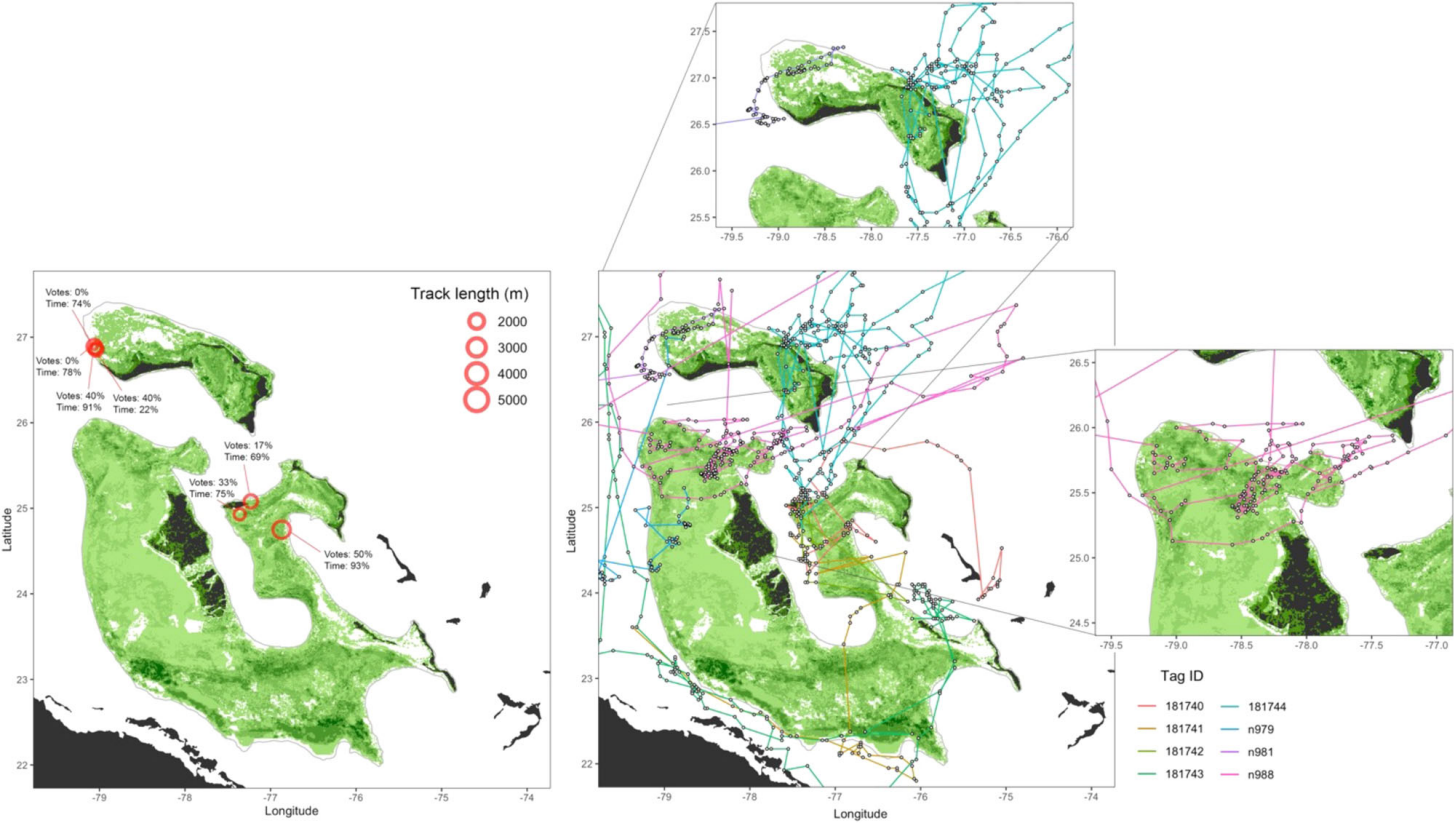
Tiger shark survey coverage on The Bahama Banks. Tracking locations from camera tags (left); Votes indicates the percentage of votes from seagrass estimates (see Supplementary Tables 1 and 2 for details) from the release location for the camera tag, and Time indicates the percentage of time the camera-tagged shark spent over seagrass. Right, locations of satellite-tagged shark tracks in proximity to the Bahama Bank, with insets highlighting highly tortuous movements in areas of dense seagrass coverage. Tag ID corresponds to individual tiger sharks.

We also tagged and tracked individual, free-swimming tiger sharks on the Great Bahama Bank and Little Bahama Bank (*n* = 15 total), with a combination of archival pop-up satellite tags to estimate geolocation and vertical habitat preferences (*n* = 8 tiger sharks equipped with satellite tags only), as well as biologging camera packages to confirm benthic habitat type (*n* = 7 tiger sharks equipped with camera tags only), including the first-ever deployment of a 360-degree camera borne by a marine animal^24^. Satellite-tagged tiger sharks traversed 4177 km on both regions of The Bahama Banks, up to 11.3 km/day, with a total of 20.6 km surveyed by sharks fitted with cameras (Fig. 3). Individual tiger shark home ranges displayed their expansive yet concentrated spatial coverage of seagrass habitat on both bank systems, as highlighted by the 95 and 50% KUDs, respectively (Supplementary Fig. 2). The average (± SE) cover of seagrass retrieved from the videos on the shark-mounted cameras was 71.5 ± 8.9%, which confirmed the strong fidelity of tiger sharks in the Bahama Banks to seagrass ecosystems^23^. Therefore, it could be assumed that the eight tagged animals not equipped with cameras spent over 70% of their time over seagrass ecosystems when they swam across shallow banks. Importantly, tiger sharks were able to scout deeper areas than those surveyed by humans (mean depth of surveys 5.4 ± 0.1 m and 16.2 ± 0.1 m for human and tiger sharks, respectively; Supplementary Figs. 4 and 5), with tiger sharks extending well below the depth limit of seagrass (maximum depth reached by tiger sharks 608 m) and further into the interior of the vast Great Bahama Bank, covering areas that were not logistically possible for human access. The greater depth reach of tiger sharks is relevant as the very clear waters on the Bahama Banks allow seagrass to grow well below normal depths accessible to SCUBA divers, with the deepest record for seagrass growth in the world reported for the neighboring Dry Tortugas (90 m)^25^.

Our prediction of the presence of seagrass across the Bahama Banks based on ground-truthed, Landsat 8 imagery generated a kernel of likelihood for seagrass presence that predicted 25,961 km^2^ of seagrass area. This estimate is conservative since we could not obtain cloud-free Landsat images in portions of the Little and Great Bahama Banks, including the entire Cay Sal Bank (Fig. 1). By integrating our prediction with existing estimates^18,19^, as well as the predicted seagrass area extracted from the recently released Allen Coral Atlas^20^ through an ensemble weighted voting system (Supplementary Tables 1 and 2), we determined that 1+ vote from all sources estimates seagrass coverage of 92,524 km^2^, 2+ votes 55,348 km^2^, and 3+ votes 23,486 km^2^ (Fig. 1). However, each estimate source spanned different areas of the banks, limiting the number of potential votes in certain regions (Fig. 1). Examining the percentage of votes from the total available in each location, >25% of votes indicate a conservative seagrass coverage of 66,990 km^2^, with an upper estimate of 92,524 km^2^ (area ≥1 vote; Supplementary Table 2). Post hoc analysis of model accuracy was performed, using an additional 126,696 seafloor images obtained via diver tows, spanning a large area of ~67 km in The Bahamas. We compared these ground-truth classifications across the predicted classifications over 120 cells from the ensemble model and found an 80% match for habitat predicted to be “seagrass” (Supplementary Fig. 3), confirming model accuracy and offering strong empirical support to our combined model integrating decades of data.

With a large range in potential seagrass values generated from each estimate source, greater insights may be gained from examining consistency with ground-truthed data. In human-measured data, >75% of sampled locations with >25% of seagrass votes contained seagrass (Supplementary Fig. 1 and Supplementary Tables 1 and 2). As another independent reference data source, camera-tagged tiger sharks spent an average of 72% of time post-release over seagrasses. In general, these areas were often predicted to contain seagrass (Fig. 3). However, in two cases there were zero votes for seagrass coverage, but sharks spent >70% of their time over seagrass, and another where there was one vote (17%) for seagrass coverage, and the shark spent 69% of its time over seagrass (Fig. 2). Hence, tiger shark habitat use signaled a consistent underestimation of seagrass area by all existing remote sensing products.

## Discussion

Our assessment of the extent of the Bahamas seagrass ecosystem builds on several data streams, including independent remote sensing products, existing and empirical, and ground-truthing supported by human observers and tiger sharks. There are two elements of innovation in our study enabling the assessment of The Bahamas seagrass ecosystem. First, the use of an ensemble voting approach allowed us to integrate estimates derived from different data streams. The second, and most innovative element, is the use of tiger sharks to extend the ground-truthing supporting the assessments. Tiger sharks, the largest apex predator in tropical seas, collected innovative data on benthic habitat in our study, serving as a unique survey partner and tool to assess seagrass extent^23,24^ and habitat configuration (sparse seagrass vs. dense seagrass). The data they derived significantly extended the scale of groundtruthing in our study area, therefore helping to overcome the challenges around ground truthing which thus far had prevented the robust assessment of the extent of the seagrass ecosystem across the Bahamas Banks. Despite the fact that we provided here the largest volume of human-based survey data for any seagrass ecosystem in the ocean, ground-truthing supported by human observers remained confined to a relatively small area and narrow depth range. Pooling tiger shark and human efforts, we assembled one of the largest and most innovative seagrass survey efforts undertaken to date, with robust ground truthing supporting the designation of The Bahamas Bank as the largest seagrass ecosystem on Earth. These findings have broad implications for estimates of global carbon storage, while highlighting the role of highly mobile animals as powerful surveyors of expansive ocean habitats.

The innovative use of tiger sharks to characterize benthic communities allowed our survey to categorize areas of habitat beyond traditional capabilities of human-based survey techniques. We found that tiger sharks surveyed a much greater depth range of seagrasses compared to humans (Supplementary Figs. 4 and 5) and covered transects averaging extrapolated distances of 51.6 km per day, compared to a few hundred meters covered by humans per day. Tiger sharks also demonstrated that remote sensing underestimated the presence of sparse seagrass areas. This was expected because much of the banks are covered by very sparse *Halophila decipiens* and *Halodule wrightii*, as detected from camera-equipped tiger sharks (Supplementary Movie 1), which are partially covered by carbonate particles and remain remarkably difficult to retrieve from remotely sensed ocean color. Further, evidence from shark and human-based surveys suggest that the actual seagrass coverage is likely toward the higher end of our estimates, suggesting that 66,990 km^2^ (>25% of votes) is a conservative estimate, with an upper estimate of 92,524 km^2^, yet enough to declare with confidence the Bahamas Banks the largest seagrass ecosystem on Earth. We infer that <30% of this area is covered by denser meadows of *Thalassia testidinum* and *Syringodium filiforme*, while most of the area is covered by the sparse *H. decipiens* and *H. wrightii*, much of which was previously undetected from remote sensing efforts alone. The research effort required to intercept and tag free-roaming tiger sharks—26 h of sampling over 10 days—was also considerably lower than the extensive time needed for human divers to perform photogrammetry transects (250 h over 30 days), for a much smaller surveyed area, demonstrating the time and cost efficiency of tiger shark surveying relative to humans. Lastly, tiger sharks proved to be better surveyors than humans, covering more linear distance per day, with the potential to cover critical seagrass areas which overlap with their home range.

The vast seagrass ecosystem of the Bahamas Banks may play a major role as a blue carbon resource (Fig. 1). Our preliminary findings from sediment coring throughout the Bahamas banks suggest carbon stocks comparable to other tropical seagrass ecosystems (0.007 ± 0.002 g C_org_ cm^−3^ for Bahamas sediments compared to 0.0067 g C_org_ cm^−3^ for tropical seagrass sediments, recalculated from ref. 26). Extrapolated down to 1 m soil depth over the likely (66,990 km^2^) and maximum (92,524 km^2^) area, we estimate the Bahamas seagrass ecosystem contains 0.46 to 0.63 Pg C_org_. This estimate represents a first order estimate of the carbon stock that may be associated with the Bahamas seagrass ecosystem. Further research is needed to better quantify and qualify this, but, relative to global seagrass carbon stock estimates^27^, our results indicate that the Bahamas seagrass may contain 19.2–26.3% of the carbon buried in seagrass sediments globally. Because the carbonate in the banks is ancient, seagrass dissolution of sediment carbon from acids and CO_2_ released by the roots and microbial respiration could potentially release important amounts of alkalinity^28,29^. Carbonate dissolution rates, and thus alkalinity release rates, have been reported to increase linearly with seagrass density in the Bahama Banks, at average rates of 7.9 mmol C m^−2^ day^−1^ ^30^, equivalent to 2.32 Tg C per year over the 66,990 km^2^ of seagrass in the Bahama Banks. Hence, carbonate dissolution in these seagrass ecosystems may greatly amplify the carbon removal potential of the Bahama Banks through alkalinity enhancement. The consensus estimates of 57,337 km^2^ of seagrass in the Bahama Banks is, however, conservative, as we note that there is a likelihood that the area may be as large as 92,524 km^2^ (1 + [<10%]) votes from available estimates). Progress to reduce uncertainty is unlikely to come from remote sensing products alone unsupported by significant parallel ground-truthing efforts, and would require a scaling-up of the animal-borne assessments used here, expanding the tagging program to sea turtles and sharks to discover putative seagrass habitat, as well as using long-range autonomous unmanned vehicles, as the area is too vast to be surveyed by divers.

Remote sensing of seagrass is a challenging endeavor; the sensor we used for our own estimate was selected for its robust temporal resolution forcing us to trade-off with a slightly lower spatial resolution, balanced by also using the estimates derived from the Allen Coral Atlas which employs the highest precision (pixel size 3.125 m) yet used to resolve seagrass from space. Further to this point, we also realize that the time periods covered by the integration of remote sensing estimates could result in changes in seagrass position or density, which may underline some of the differences between estimates derived using remote sensing images acquired in different years. However, Caribbean seagrass ecosystems are tolerant of hurricanes and the extensive carbonate bank habitat where they reside functions to dissipate and distribute wave energy from storms^31^. As a result, surveys have suggested that seagrasses in The Bahamas are relatively stable, seeing negligible changes in the last 10–20 years. Indeed, provided the vast extent of The Bahamas seagrass ecosystem resolved here (57,337–92,524 km^2^), and the challenges to accurately resolve the extent and condition of deeper seagrass components through remote sensing or human surveys, we contend that instrumented tiger sharks remain an essential approach to monitoring this vast ecosystem.

Large, highly mobile marine megafauna should be viewed as important collaborators in future research and conservation efforts surrounding the discovery and protection of key blue carbon hotspots in the ocean, including monitoring the dynamics of The Bahamas seagrass ecosystem. Through critical ground-truthing of seagrass extent by camera-equipped tiger sharks, we were able to designate The Bahama Banks as the largest seagrass ecosystem on the planet. This region is likely to be a globally relevant sink for atmospheric carbon, and likely plays a key role in protecting the shallow carbonate bank, the islands, and the beaches (that support much of Bahamas tourism income) from erosion under the increasing cyclones impacting the region. The Bahama Banks seagrass ecosystem also supports important fisheries, which target commercial species such as the Nassau grouper *Epinephalus striatus*, Queen conch *Lobatus gigas*, Caribbean spiny lobster *Panulirus argus* and other commercial species that reside in seagrass ecosystems during part of their life history^32^. Protecting this seagrass ecosystem and taking action to remove pressures is, therefore, imperative and could be the basis for an important blue carbon strategy for The Bahamas to contribute to climate action and economic resilience. Aragonite mining, under a moratorium for the past 40 years, and dredging for coastal infrastructure and development may be the most significant threats to this seagrass ecosystem. The long-term protection offered to tiger sharks in The Bahamas has likely played a role in keeping seagrass habitat and herbivores in an ancient and natural balance, while also facilitating their support as allies to explore and monitor this expansive ecosystem.

## Methods

### Ground-truth surveys of seagrass habitat

To obtain georeferenced field data on benthic cover levels from habitats of the Bahama Banks, we employed two similar, in-water survey and image approaches: (1) swimmer-based photo-transects; and (2) tow board photo transects (Supplementary Fig. 6), resulting in a total of 2542 surveys.

For (1), free-divers swam over the bottom of the seafloor at a fixed height with a digital camera (Canon 5D mIV, GoPro Hero) set to capture images manually. Photographs were captured using automatic settings in a 1.0 m × 1.0 m footprint, 1.5 m above the seafloor following [39]. A center console vessel was used to run the transects at distances of 5–7 km, whereby the free-diver would capture successive photos at a horizontal distance of between 400–800 m, and the location was logged using either a handheld GPS (Garmin GPS 73) or a boat-mounted GPS with a depth sounder (Garmin EchoMap DV). Transect locations were chosen based on a priori local expert knowledge of varying benthic cover in the region. Surveyed areas included: southern New Providence (24.948862°, −77.387834°), southeast of New Providence (24.980265°, −77.229168°), south of Rose Island (25.066268°, −77.160063°), the middle Great Bahama Bank (24.735355°, −77.212998°), and the northern Exumas (24.729973°, −76.889488°). For (2), snorkeling observers were pulled from a research vessel on tow boards affixed with underwater action cameras (GoPro Hero 3+) traveling at ~1 m/s. The start and end of a tow were delineated with either a handheld GPS (Garmin eTrex 30) or a boat mounted GPS with depth-finder (Garmin EchoMap DV), and tows proceeded in a straight line recorded by the GPS. Cameras recorded images at 0.5 Hz throughout the tow, starting in conjunction with creating a waypoint. Samples (i.e., paired image and geolocated point) were sub-selected from the tow once movement began, at the midpoint of a tow, and immediately before movement stopped. Images were manually quality controlled such that if a selected image contained obstructions or was out of focus, the nearest clear image was selected to replace it. If no images within 10 s were clear (i.e., 10 m maximum spatial error), the sample was discarded. If the GPS track contained gaps or segments larger than 10 m, only images/point pairs at the start and end waypoints were sampled.

Surveys focused on historical fishing grounds for queen conch (*Lobatus gigas*) between 2015 and 2018 following the sampling design and methods of ref. 32. A stratified random design was used to allocate 6000 m^2^ of observation effort into each cell of a 1’ by 1’ grid placed over each fishing ground. This effort was split into multiple tows between 200 and 1000 m in length, thus images were separated by at least 100 m.

Fishing grounds extended from the edge of a deepwater sound to between 7 and 10 km up the bank and were limited to the depths used by freediving fishers. Surveyed fishing grounds included: the Exumas (24.382207°, −76.631058°), the southwestern Berry Islands (25.455529°, −78.014214°), south of Bimini (25.375592°, −79.187609°), the Grassy Cays (23.666864°, −77.383547°), the Joulter Cays (25.321297°, −78.109251°) and the southeast tip of the Tongue of the Ocean (23.376417°, −76.621943°). For details on image processing, see section on remote sensing below.

### Sediment coring

To gather the sediment cores analyzed for organic carbon content on the Bahama Banks, we collected samples from various benthic habitats that included varying densities of seagrass habitat (*Thalassia testidinum* and *Syringodium filiforme)*. We percussed, via SCUBA, an acrylic cylinder tube perpendicular to the seafloor into marine sediment until rejection at various penetration depths up to 30 cm. The sample was then extracted vertically from the marine sediment and capped at the bottom to avoid loss of material. This sample was then transported vertically through the water column to a research vessel where it was removed from the coring device and immediately capped on top with an air-tight cap. Compression rates were negligible (~5 cm) across the first 5 cores, and as such were not subsequently measured. The samples were then labeled, photographed, geotagged, and the first 30 centimeters of each core was extruded. To complete the extrusion process, we placed each sample on top of a capped piston device in the same orientation as collection (deepest portion of collected sediment still on the bottom). The bottom cap was removed to thread the acrylic cylinder tube onto the piston device and then was lowered to various measured lengths to collect corresponding depth sections of the sediment core. These sections were sliced (every 1–5 centimeters), labeled, and placed into whirl pack bags to collect the wet weight of each sample. All samples were then frozen and stored for future laboratory analyses. All samples were dried in a laboratory oven at 55 °C for 48 h until constant dry weights were reached. The samples were then weighed to collect their corresponding dry weights. The dry bulk density (DBD) was calculated by diving the sample dry weight (g) by the sample volume (cm^3^). The samples were then further ground with a mortar and pestle until a homogeneous fine grain size was achieved. Sediment samples collected from the Exuma Cays (142 samples from 16 cores) were analyzed for Corg content. Sediment samples were weighed accurately into silver capsules and acidified with 4% HCl until no effervescence was detected in two consecutive cycles. The samples were then dried in a 60 °C oven overnight, encapsulated into tin capsules and analyzed using an Organic Elemental Analyzer Flash 2000 (Thermo Fisher Scientific, Massachusetts, USA). We then conducted a standard loss on ignition (LOI) methodology at our laboratory facility (Braintree, Massachusetts, USA) for all the samples. Each sample was subsequently sub sampled with 5–15 grams of representative material and placed into a ceramic crucible to collect its mass. The crucibles were then loaded into a separate muffle laboratory oven and heated at 550 °C for 6 h. Upon completion of this muffle, the crucibles were then immediately weighed to collect the LOI of organic material from each sample, defined as the weight lost in the muffle (g) divided by the subsample dry weight (g). A fitted regression between the Corg and LOI from the Exuma Cays cores was generated (Supplementary Fig. 7), and then used to predict the sediment Corg contents from LOI measurements in the Grand Bahama cores. Sediment Corg stocks were quantified by multiplying Corg and DBD data by soil depth increment (1–5 cm) of the sampled soil cores. The cores from the Exuma Cays (15 cm) and Grand Bahama (30 cm) were collected with different depths, we therefore fitted a regression between Corg stock in 15 cm-depth and Corg stock in 30 cm-depth for the Grand Bahama cores (Supplementary Fig. 8) and used this regression to extrapolate Corg stock of the Exuma Cays cores into 30 cm-depth. Moreover, to allow direct comparison among other studies^27^, the Corg stock per unit area was standardized to 1 m-thick deposits by multiplying 100/30.

### Tiger shark tagging

The research and protocols conducted in this study complies with relevant ethical regulations as approved by the Carleton University Animal Care Committee. The shark data used in this paper were collected as part of a multi-year, long-term research program evaluating the interannual behavior and physiology of large sharks throughout the coastal waters of The Commonwealth of The Bahamas^23^. All sharks were captured using standardized circle-hook drumlines^33^ on the Great and Little Bahama Banks throughout the country, focusing efforts in three primary locations: off New Providence Island, the Exuma Cays, and off West End, Grand Bahama, from 2011–2019. All sharks were secured alongside center console research vessels and local dive boats, where their sex, morphometric measurements, and blood samples were taken. A mark-recapture identification tag was applied to the shark at the base of the dorsal fin. Some of the sharks sampled in the present study were also tagged with a coded acoustic transmitter which was surgically implanted ventrally into the peritoneal cavity and then sutured, as part of a concurrent study on shark habitat use and residency within the region^23^.

Pop-off archival satellite tags were affixed to eight tiger sharks (seven female, one male; 298 ± 28 cm total length; mean ± SD) in The Bahamas from 2011–2019, permitting measurements of swimming depth and water temperature recorded at either 4-min (Sea-Tag MODS, Desert Star Systems LCC, USA) or 10-s intervals (miniPAT tags, Wildlife Computers, USA). Pop-off satellite tags were inserted into the dorsal musculature of the sharks using stainless steel anchors and tethers. All pop-off satellite tags were either recovered manually, permitting access to the full time-series, or popped-off and transmitted their data to an Earth-orbiting Argos satellite, resulting in a subset of the full time-series (transmission frequencies: 2.5 min [miniPAT], 10 min [PSATGEO], daily average [Sea-Tag MOD]). Tiger shark positions were estimated from the satellite data using tag-specific proprietary state space algorithms from Wildlife Computers (GPE3; based on ref. 34) and Desert Star Systems^35^. With miniPAT tags, positions were further filtered to remove the least reliable positions (<0.1 observation score). Tracking durations with reliable positioning estimates were variable (mean *=* 144 days; 44 to 376 day range). Descriptive statistics of depths experienced by sharks (*n* = 176,206) were generated and depth use patterns were plotted for the periods where reliable positioning data were available (Supplementary Figs. 4 and 5). Shark satellite positions were filtered to include just the positions located on the Bahamas Banks. Kernal utilization distributions (KUD) were calculated using the adehabitatHR package^36^, from which 95 and 50% KUD polygons were extracted. This was conducted with satellite tracking data from 5 sharks for which there were sufficient datapoints for this analysis.

Camera tag biologger packages were affixed to a subset of 7 mature tiger sharks on the Great and Little Bahama Banks from 2016–2020, using two methods: (1) capture and release using hook and line, following the same methods as above; and (2) in-water placement on free-swimming tiger sharks. In (1), we built custom camera-tag packages using a positively buoyant material (Diab Syntactic © non-compressible foam). All cameras used were forward-facing, and uniidirectional, with the exception of one unit which was a 360-degree camera^24^. Two asset recovery tags were secured to the center of the biologger payload using clear silicone: a satellite tag (SPOT-386A, Wildlife Computers, Redmond, WA, USA) and a VHF radio tag (F1840B, Advanced Telemetry Systems, Isanti, MN, USA). Two stainless steel nuts were added to the package to provide forward-facing ballast to reduce the buoyancy from the camera housing, thus allowing the tag to float on the surface in a manner which maintained the vertical orientation of the satellite and radio tag antennae in air. The entire biologger package was attached to the left side of the shark’s dorsal fin by drilling two small holes and threading two connected, biodegradable cable ties through and around the package. The heads of the cable ties were then joined together via the eyes of a dissolvable galvanic timed-release swivel (A2 model, Neptune Marine Products, Port Townsend, WA, USA), which would eventually corrode in seawater after an estimated period of ~24 h (swivel was pre-dissolved to permit a short-term deployment), thereby allowing the positive buoyancy of the package to cause it to naturally release and come off the animal. Once attached, the camera was activated for recording and the shark was released. The entire time to collect all animal data, apply tags and attach the biologger was 12 min. In (2), small action cameras (GoPro Session) were inserted into custom float packages attached to stainless-steel clamps and were placed firmly on the dorsal fins of free-swimming tiger sharks. Dissolvable galvanic swivels were used as above, programmed to corrode after ~6–12 h. A single VHF transmitter tag as above was included in the package to aid in asset recovery.

Collectively, camera tag deployments ranged from 55.43–117.06 min of recorded footage, with a mean of 80.81 ± 11.65 min (Additional Supplementary Information). All camera tags were ultimately recovered by traveling to the most recent ping from the satellite tag while actively using a VHF radio to locate the pings from the VHF transmitter. Swimming speed estimates were obtained from the camera tag-packages by visual inspection of the footage and conversion from tailbeat frequency (TBF). Footage was played back at 1.5x speed, with the sharks’ head in the lower center of the screen. To estimate TBF, an observer manually tallied the number of times the shark moved its head from the right-hand side of the screen to the left-hand side and back to the right-hand side, counting this as one tailbeat. The total number of tailbeats per minute was recorded. Speed (*S*; m s^−1^) was estimated as *S* = SL*TL*TBF, where *SL* is average stride length, 0.36 body lengths/tail beat, which was digitized and interpolated from a previous study on tiger sharks^37^, and *TL* is total length in m. This was also used to calculate the total distance traveled by each shark. Classification of time spent over seagrass habitat was determined by counting the cumulative duration (minutes) when each tiger shark was seen swimming over any type of seagrass habitat, regardless of species or density. Total percentage of time over seagrass was then calculated for each shark by dividing time by the total track length (minutes), and the geolocation where each animal was tagged and released was used for spatial reference.

### Empirical remote sensing

Remote sensing was used to map benthic seafloor habitat extent across the large spatial scales as seen in the study area^38–40^. Seven Landsat 8 (OLI) tiles (path/row, see Supplementary Table 3) covering study sites around The Bahamas were used in this study. Landsat 8 was chosen as it has among the best potential to facilitate large-scale seagrass density mapping due to its strong temporal resolution and algorithms for water-based corrections. Landsat has equivalent spectral characteristics to many other sensors (e.g., Sentinel-2, Hyperion, SPOT, ASTER, CBERS). We recognize Landsat has slightly lower spatial resolution than Sentinel-2. Images from 2019 and 2020 which had minimal to no cloud over near shore areas, which fell within the depth range of seagrass species, were chosen from web server of the United States Geological Survey (USGS; https://earthexplorer.usgs.gov/). More tiles would have been obtained; however, quality cloud-free images were not available for certain areas, such as the southern Bahamas. It was assumed that there were no significant changes between image acquisition years (less than two; Supplementary Table 3) and file data collection periods. Earth Resources Observation and Science ortho-rectified and terrain corrected (L1T) all Landsat imagery (https://www.usgs.gov/core-science-systems/nli/landsat/landsat-levels-processing). The visible (red, green and blue) and near-infrared bands, with 30 m spatial resolution of OLI data, were involved in cloud detection and land mask, while only water penetrating visible bands were used for seagrass mapping. Prior to seagrass mapping, the *Fmask* cloud detection algorithm^41^ was used for masking clouds, shadows, and land cover from each Landsat tile. Next, all raw OLI images (visible bands) were converted into top-of-water reflectance for radiometric correction following standard procedure suggested by the USGS (https://www.usgs.gov/core-science-systems/nli/landsat/using-usgs-landsat-level-1-data-product). For this study, the simple Dark Object Substract 1 (DOS1) atmospheric correction method plugin^42,43^ was implemented in the QGIS (v. 3.18). A water column correction^44^ was applied to radiometric and atmospheric corrected imagery. The depth invariant bottom index retrieved from the bi-plot of the reflectance of visible band-pairs^15^, for blue-red, red-green and green-blue, were layer stacked. Finally, all tiles were joined in a mosaic and cropped a subset of 170,388 km^2^ prior to seagrass classification. Excluding cloud, shadows, and land, about 88,000 km^2^ of ocean area were used for seagrass cover mapping.

A total of 2542 georeferenced, in situ field photos were collected uniformly along the bank of the Bahamas. The dominant benthic vegetation species belonging to the field photo were identified by expert knowledge. Seagrass cover percent for each photo was precisely estimated using image thresholding technique available in ImageJ (v. 1.53e)^45^, a java-based image processing software (https://imagej.nih.gov/ij/). All color (RGB) photos were converted into 8-bit grayscale prior to thresholding. Sand cover can easily be determined through automatic thresholding technique, from where fraction seagrass and non-seagrass cover can easily be determined through substruction of sand cover from total area of each photo. All field data thus were divided into four seagrass cover-types (I represents <25%, II represents <50%, III represents <75%, and IV represents <100% seagrass cover), non-seagrass and submerged sand substrates (Supplementary Table 4). Half of field data were used for training OLI data and half for accuracy assessment. Whereas the 1 m × 1 m ground-truthing images were much smaller than the 30 m × 30 m pixel size of the remote sensing product, we found that adjacent pixels tended to be homogeneous as spatial variability in seagrass configuration is small along the Bahamas Banks where environmental gradients shaping seagrass ecosystems are smooth (Supplementary Fig. 6).

We then used machine learning neural network (NN) algorithms^46,47^ for seagrass classification, which was carried out in ENVI (v. 5.3). For the NN algorithm, the key parameters optimum for classification of seagrass cover classes were: training threshold contribution (0.8), training rate (0.1), momentum (0.9), and number of hidden layers (1 for non-linear). A confusion matrix was calculated using ground truthed ROIs^47^ to assess the accuracy of NN parameters. The accuracy measures^48^ were expressed in terms of overall accuracy, producer’s and user’s accuracies, and kappa coefficient. The accuracy of classified Landsat 8 images produced by using NN analysis showed evidence for misclassification between individual seagrass density classes, thus yielding a relatively acceptable (70.2%) overall accuracy; however, “seagrass” was correctly mapped (Supplementary Table 5). Consequently, the Kappa value was also found to be low (0.6, i.e., <0.8), which was expected for mapping a large region like The Bahamas, at the spatial resolution of Landsat (30 m). Lower user’s (40%) and producer’s (56%) accuracies were achieved for the discrimination of sparse “Sg-I” class (<25% cover). Higher accuracies were recorded for medium density “Sg-II and III” classes, compared to the highest density “Sg-IV” class. The NN classifier can be considered effective since a lower number of predicted seagrass class pixels were assigned as either non-seagrass or sand pixels (Supplementary Table 6), resulting in an acceptable seagrass cover class map without over- or under-estimating seagrass cover areas for The Bahamas.

### Comparison of remote sensing products

The current mapping efforts were integrated with several previous seagrass estimates^18–20^ to generate an integrated, composite range of estimates of seagrass coverage on The Bahamas Banks. This approach did two key things: (1) compared overlaps from each remote sensing product, to generate estimates of agreement on seagrass habitat; and (2) generated composite maps and calculated the spatial extent predicted from each category. This resulted in a range of estimates, from high to low probability. Seagrass shapefiles from ref. 20 were downloaded from the Allen Coral Atlas website (https://allencoralatlas.org/atlas/#5.39/24.3807/-76.0918). This product reported good accuracy for Caribbean seagrass (up to 67% match between observed and predicted), although it used ground-truthed sample images from The Dominican Republic and US Virgin Islands for their Bahamas predictions, which is not authentically representative. From empirical products^18,19^, seagrass estimates were derived from images (Fig. 1). Images were georeferenced using raster georeferencer in QGIS (Version 3.12.3). The remainder of analyses were conducted with R^49^ via RStudio^50^. Color bands were used to extract the seagrass estimates from each image ([19]—greyscale values 80–120 were assigned to seagrass; [19]—green values ≤ 170 dense seagrass [>70%], red bands ≤ 145 sparse seagrass [<70%]). Bands were determined by visual inspections for consistency with existing images.

To generate an overall weighted estimate of seagrass distribution, estimates from current mapping^18,19^ were integrated into a standardized raster grid. The grid was generated across the entirety of the banks with a resolution of 0.01° lat/lon, producing cell sizes of 1.23 km^2^. The resulting products had similar estimated seagrass extents to those reported in empirical research ([18] = 65,436 km^2^, estimated = 63,841 km^2^; [19] = 37,000 km^2^, estimated = 33,952 km^2^). Estimates from the three sources were used to generate an ensemble seagrass distribution estimate via a weighting scheme, where dense seagrass from current estimates (>50% seagrass) and dense seagrass from [18] (>70%) were given two votes, and low density estimates from these sources, as well as seagrass (density unspecified) from [19] were assigned one vote (Supplementary Table 2). Estimates from each source spanned varied regions of the banks; therefore, for each raster cell, the percent votes from the total available were calculated (Supplementary Table 2). Various cutoff percentage points were used to estimate the potential seagrass coverage on the banks based on the weight of evidence.

### Post hoc analysis of ensemble model accuracy

Classifying linear habitat tows: Image streams of the benthos were collected by action cameras mounted beneath tow boards operated by skin diving observers in search of queen conch in The Bahamas. Images were taken at a frequency of 2 Hz. Boards were dragged at between 0.5 and 1.5 m/s depending on sea surface conditions and at a relatively constant speed chosen by the observer at the start of each tow. The start and end locations of each tow were marked via GPS with a resolution of ~2 m. The continuous nature of the image stream which included shifts in visibility and depth and caused a constantly fluctuating view of the benthos made categorical assignments qualitative. The dominant habitat type and approximate coverages were estimated from an initial view of the benthos, and then major habitat transitions were noted in subsequent images. Here we define a transition as a notable shift in habitat type (i.e., seagrass to sand) or coverage (i.e., 25 to 75% seagrass coverage) that persisted for more than 10 images (~5 m of bottom). At each transition, the sequence of the image was noted along with the new habitat type(s) and coverage(s). We used the same seagrass categories as those used in our remote sensing satellite imagery model: SG-I (>0 and <25%), SG-II (<50%), SG-III (<75%) and SG-IV (≥75%). We further divided the NSg category into C-I (<25% hard substrate with living coral), C-II (>25% hard substrate with living coral), MA-I (<25% macroalgae), MA-II (>25% macroalgae), G-1 (<25% gorgonian plain), G-II (>25% gorgonian plain) and included a hardbottom category for pavement. Only living benthic coverage was classified (i.e., random seagrass blades that were not rooted were not counted toward coverage). The same observer (A. Kough) classified all the habitat, had been a participant in collecting tow data at all sites and was familiar with how the benthos appeared both on camera and in person.

Comparing linear ground-truth data to integrated, ensemble model: Tows proceeded linearly at an approximately constant velocity thus position could be inferred from image order in between two geolocated points. For example, on a tow with 1200 images, image 300 corresponded with a location 25% between the start point and the end point. Points for each transition were calculated via image order and lines containing the habitat classes were created as shapefiles in ArcGIS. The rasterized ensemble estimate, which integrated four remote sensing estimates (including our own empirical estimate), was transformed into a polygon layer and then a georeferenced intersect was calculated between the Ensemble polygons and the habitat tow lines in ArcGIS. Benthic classes from the tow data were reassigned into seagrass (SG) or not seagrass (NSG) to correspond with the Ensemble’s predictions.

We chose a random subset of 197 tows covering 69 km from available data in the Berry Islands, Grassy Cays, Exuma Cays, Bimini and Jolter Cays to classify for ground-truthing. This resulted in an analysis of 126,969 additional benthic images, which fell into 97 cells within the ensemble model, with a mean of 2.5 tows/cell. Ultimately, we obtained 909 segments of the benthos along the tows and split by the Ensemble cells. Each cell was assigned the class of the Ensemble prediction, as our goal was to verify the voted classification. If part of a ground-truthing tow fell within a cell and contained any of the habitat type that matched the Ensemble prediction, it was considered a plausible verification of the classification. In addition, we calculated the ratio of the amount of towed distance that matched the Ensemble’s prediction against the total towed distance within each cell as a measure of agreement. Results suggested that the Ensemble’s predictions were verified by ground-truthing in most cells (~80%, Supplementary Fig. 3). For example, 83 of the Ensemble cells containing tow data for ground-truthing were classified as SG. Tow data verified that 65 of these cells contained areas dominated by seagrass. Further, in 50 of these cells most habitat encountered in the tows was seagrass.

### Reporting summary

Further information on research design is available in the Nature Research Reporting Summary linked to this article.

## Supplementary information

Supplementary Information

Description of Additional Supplementary Files

Supplementary Movie 1

Reporting Summary

## Data Availability

All empirical data and derived estimates used in the analyses are accessible through DRYAD^51^. The tiger shark tracking data and camera tag results from this study have been deposited in the DRYAD^51^ data repository. The seagrass mapping data, processed seagrass estimates and ground-truth seafloor photogrammetry information from this study are available DRYAD^51^ data repository. The Allen Coral Atlas mapping data were downloaded from: https://allencoralatlas.org/atlas/#5.39/24.3807/−76.0918. Source data are provided with this paper.

## Source data

Source Data

## Code availability

All code can be accessed in the DRYAD^51^ data repository listed above, or can be made available by contacting the authors directly, where deemed appropriate.
