# Supplementary material for: Tiger sharks support the characterization of the world’s largest seagrass ecosystem
Source: Nat Commun. 2022 Nov 1;13:6328. doi: 10.1038/s41467-022-33926-1 (PMC9626626; doi:10.1038/s41467-022-33926-1)
Supplement: Supplementary file 1 — Supplementary Information [file 41467_2022_33926_MOESM1_ESM.pdf]

Supplementary Information. Supporting information to Gallagher et al. (2022). Tiger sharks support the characterization of the world's largest seagrass ecosystem. *Nature Communications.*

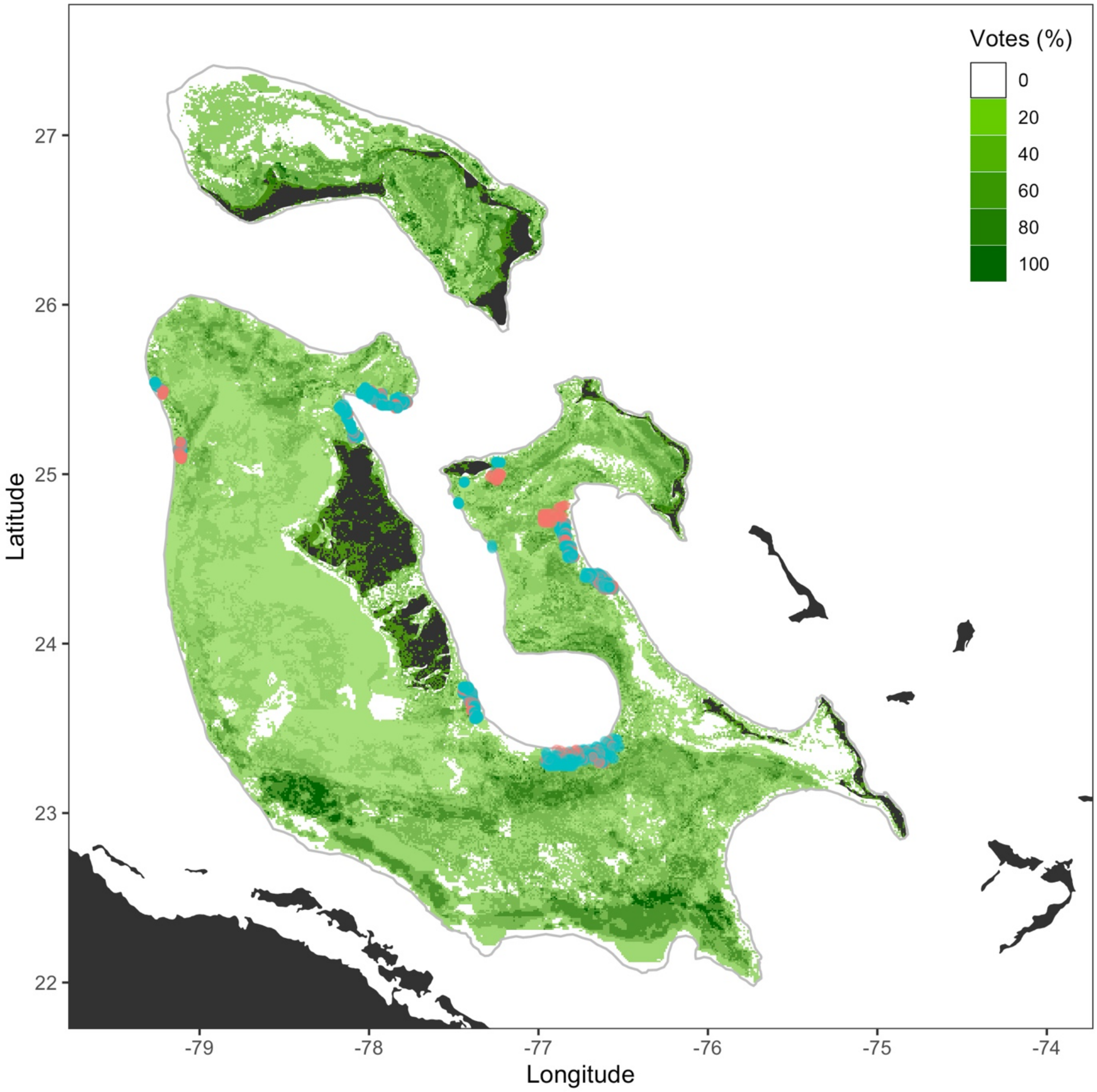

Supplementary Figure 1. Map of ground truth sample locations where human researchers collected images of benthic coverage. Samples are grouped as seagrass (blue) or sand (pink), with the total projected seagrass extent (green), as derived from the combined remote sensing estimates. Sample points are larger than true points to allow detection in the image. These surveys covered dense and sparse seagrass meadows, as well as bare, sandy areas.

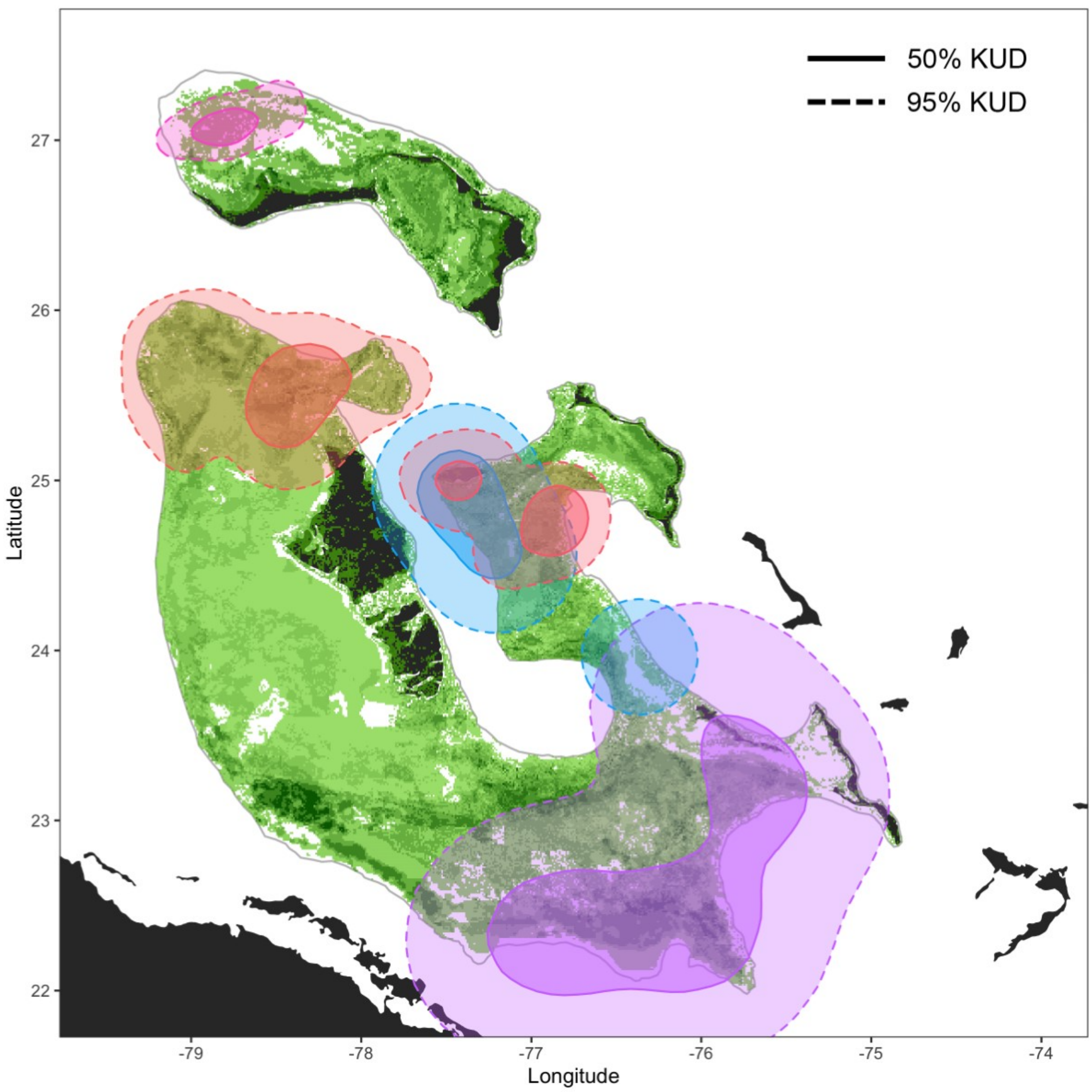

Supplementary Figure 2. Kernel utilization distributions (solid line=50% KUD, dashed line=95% KUD) for five sharks tracked with satellite tags with sufficient data for this analysis on the Bahamas banks.

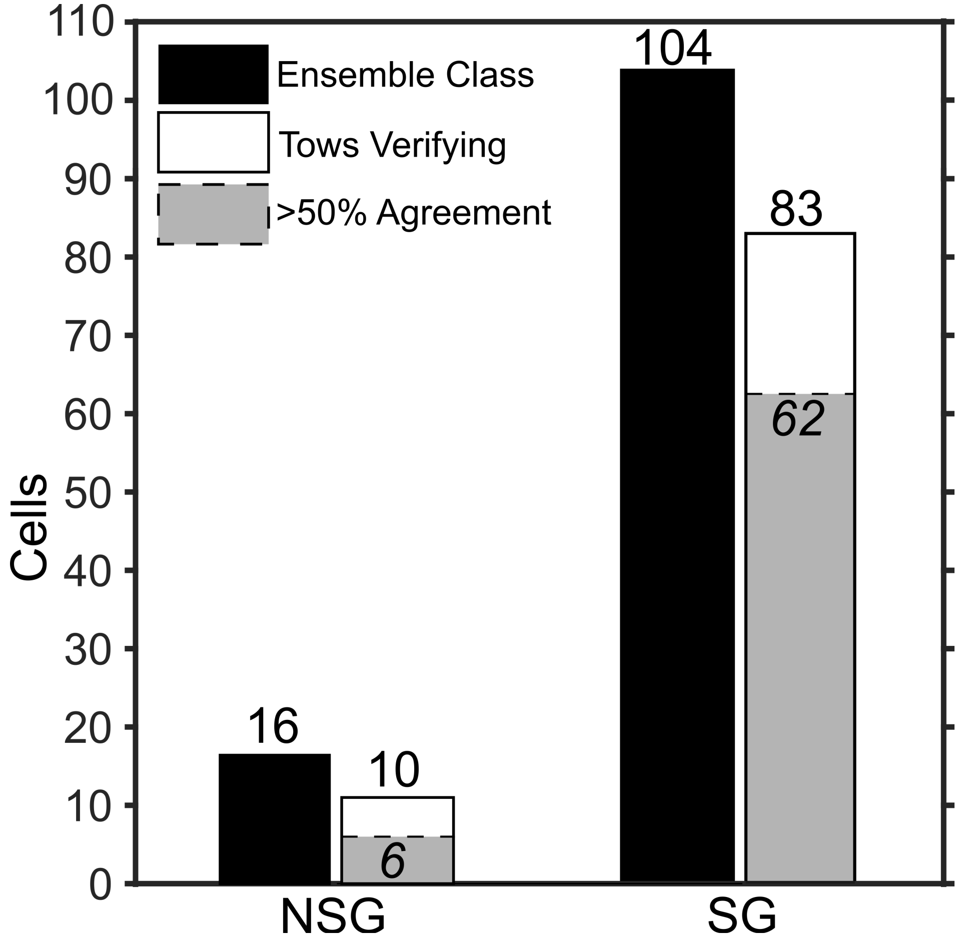

Supplementary Figure 3. Ground truth of ensemble predictions of seagrass. Three remote sensing models of Bahamian seagrass extent were integrated to determine likely coverage on a 0.01˚ × 0.01˚ cell size grid. Manually classified image streams from cameras capturing the sea floor during tow surveys behind skiffs in shallow water enabled a confirmation of cells that contained seagrass (SG) and non-seagrass (NSG) habitats (white) as predicted by the ensemble (black). Tows where more than half of the habitat surveyed matched the ensemble prediction (grey) offer strong empirical support to a combined model that integrates decades of data. Source data are provided as a Source Data file.

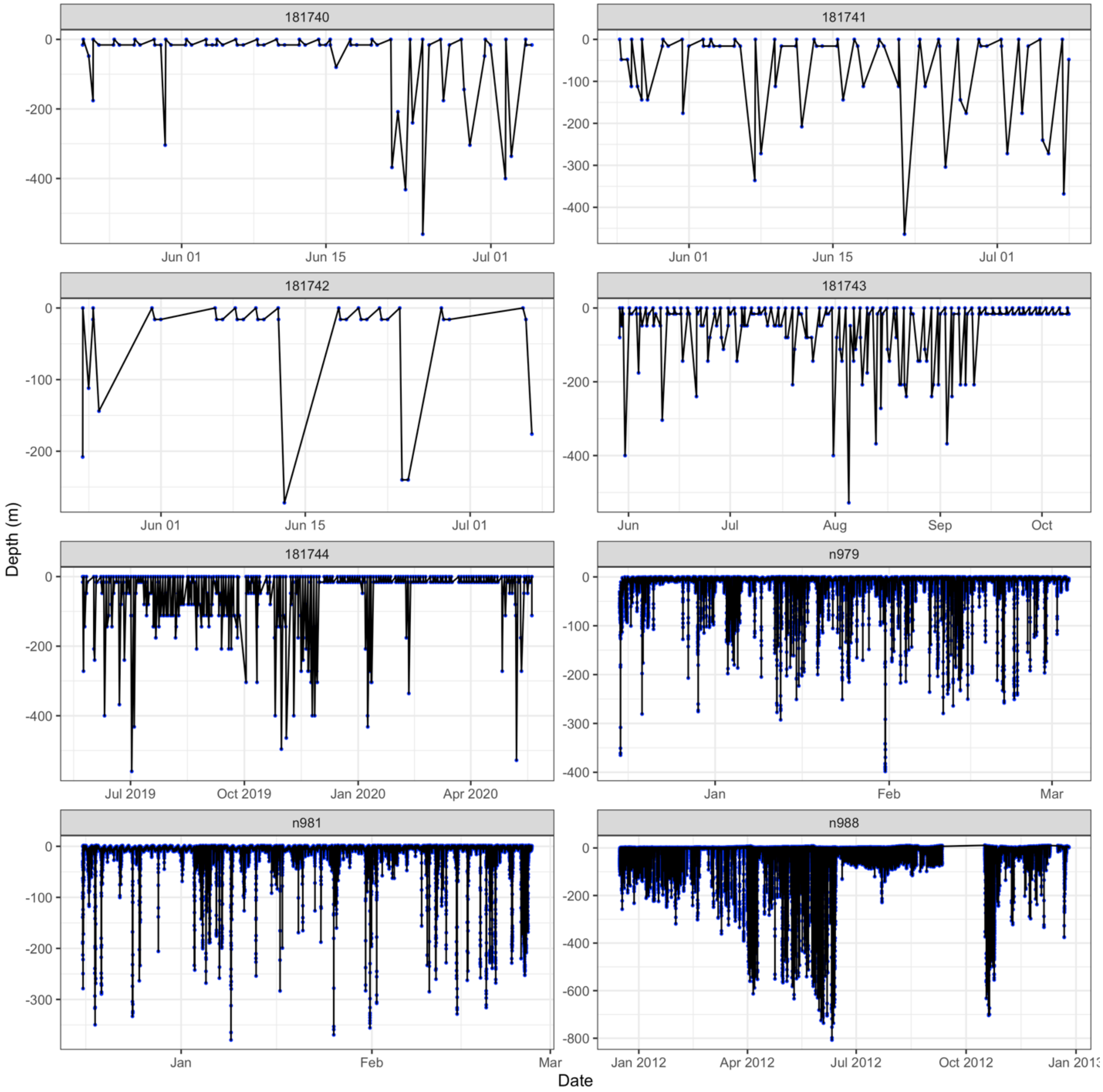

Supplementary Figure 4. Diving behavior of tiger sharks. Depths (meters) recorded from satellite tagged tiger sharks in proximity to The Bahamas Banks. Each panel represents a unique individual shark, with the time-stamp for each record on the x-axis. Source data are provided as a Source Data file.

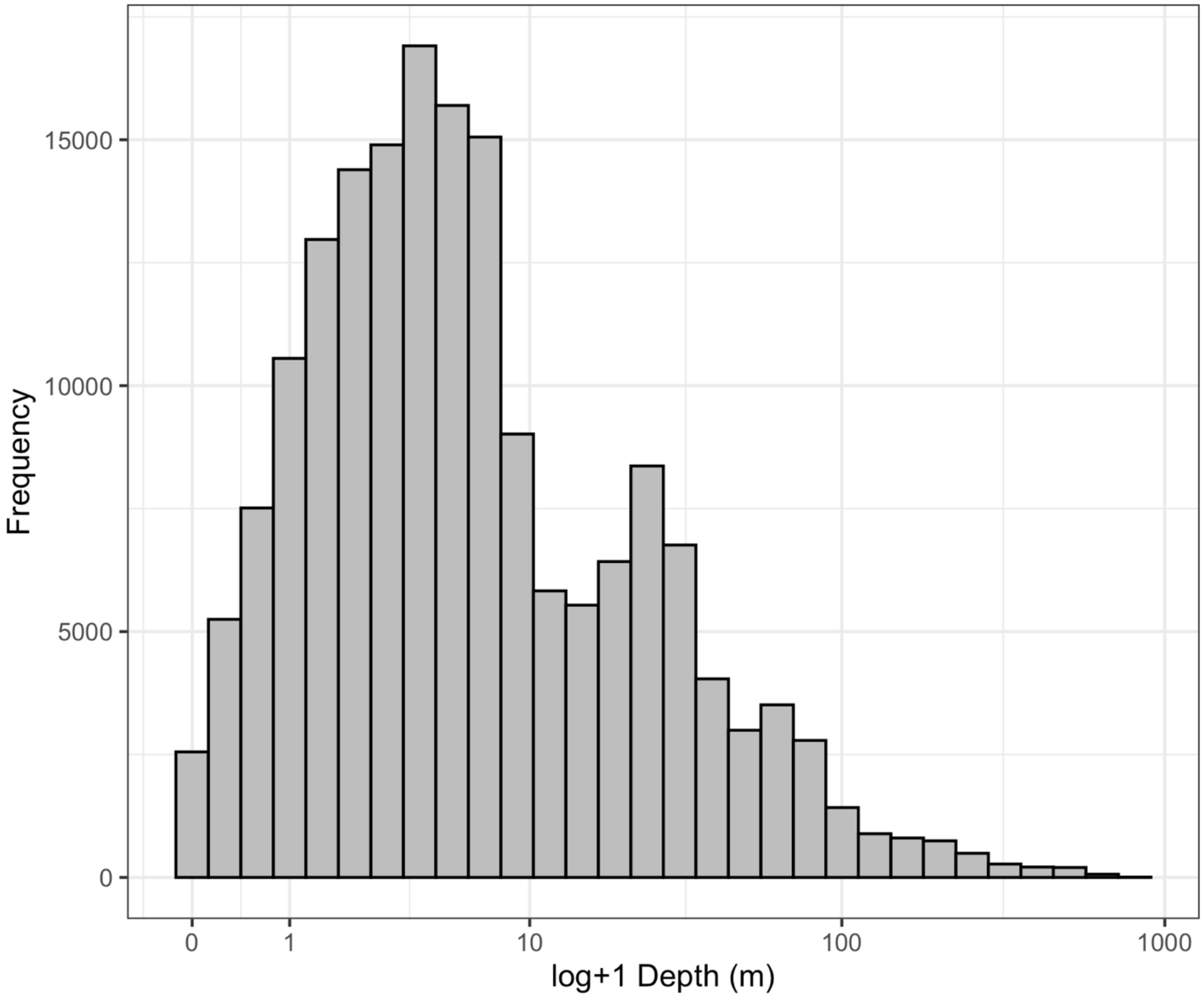

Supplementary Figure 5. Frequency histogram of swimming depths of satellite tagged tiger sharks in proximity to The Bahamas Banks. Plotted data represent pooled depth readings from tiger sharks (n = 8) equipped with pop-off satellite tags from 2011 – 2018. Source data are provided as a Source Data file.

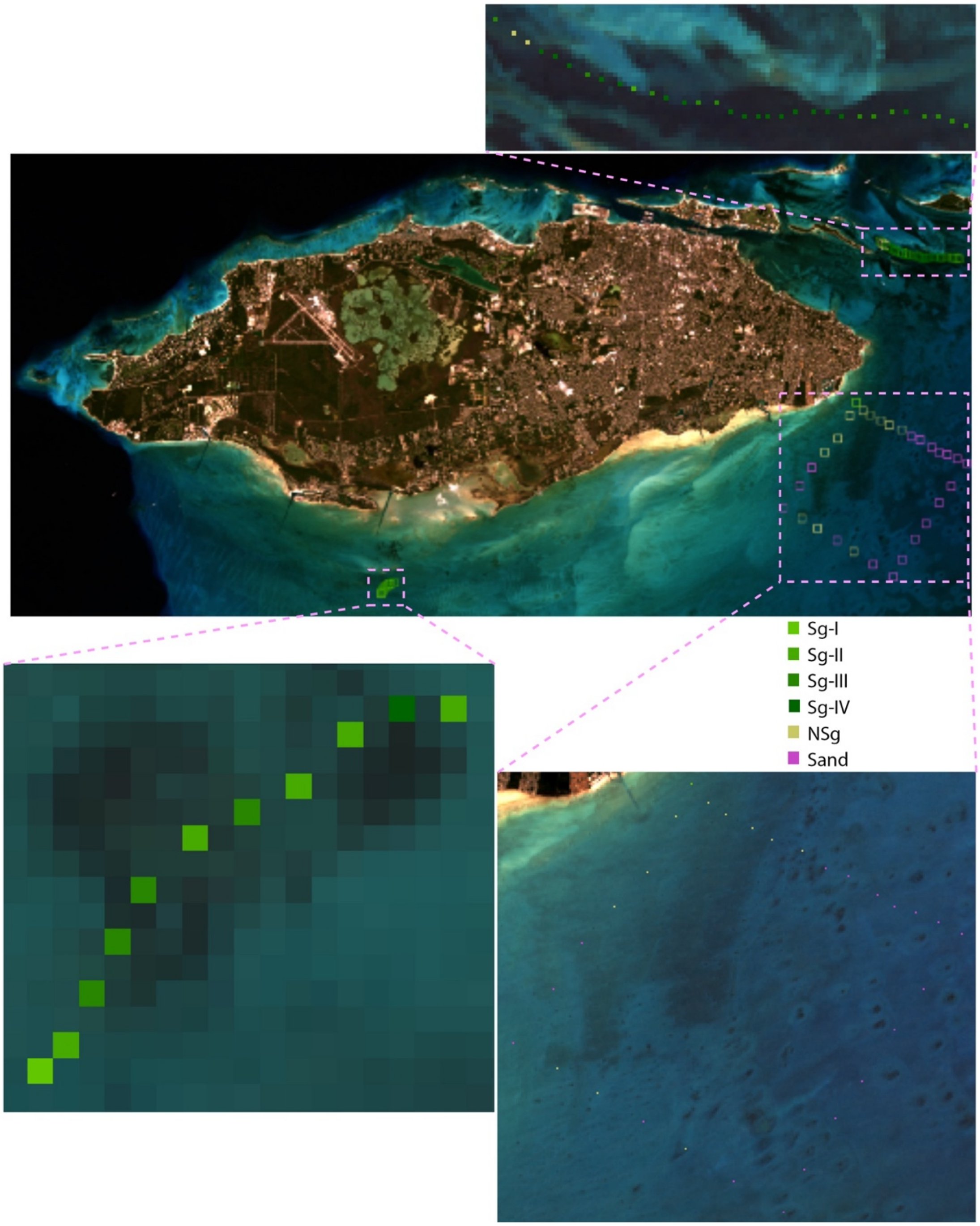

Supplementary Figure 6. Example transects used for seagrass density data collection from the near-shore areas of The Bahamas, specifically the Great Bahama Bank south of New Providence Island, illustrating reliable spatial correlation in seafloor type between field photos (1 x 1 m) that were assigned as corresponding Landsat 8 OLI pixel (30 x 30 m). Colors of squares represent different seafloor types, according to the legend.

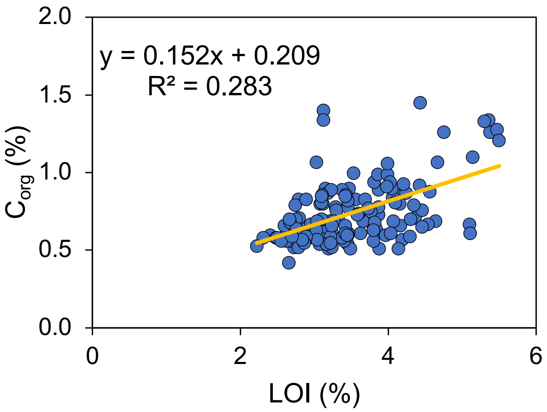

Supplementary Figure 7. Relationship between LOI and C_{org} for seagrass sediments from Exuma Island, The Bahamas (n=142). Source data are provided as a Source Data file.

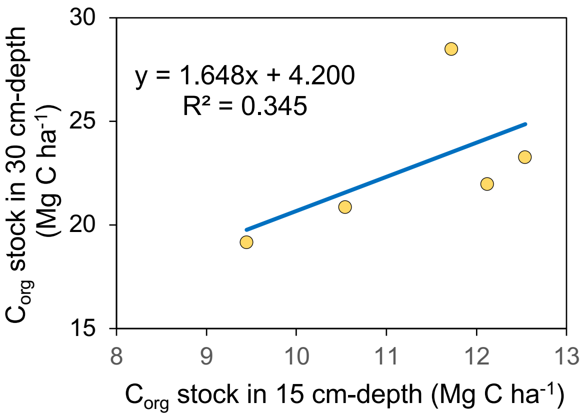

Supplementary Figure 8. Correlations between C_{org} stock in 15 cm-depth with C_{org} stock in 30 cm-depth for Grand Bahama cores (n=5). Source data are provided as a Source Data file.

Supplementary Table 1. Votes system used to generate a weighted estimate of seagrass coverage on The Bahamas Banks based on empirical and current mapping estimates.

Source	ID	Votes
(18)	seagrass <70%	1
(18)	seagrass >70%	2
(19)	seagrass	1
*	Sparse seagrass	1
*	Dense seagrass	2
Current (2022)	seagrass <50%	1
Current (2022)	seagrass >50%	2

Supplementary Table 2. Seagrass coverage based on all remote sensing products.

Votes	km^2	Votes (% total)	km^2
0+	112329	0+	112329
1+	92524	10+	92524
2+	55348	25+	66990
3+	23486	50+	30234
4+	6424	75+	6042
5+	924	90+	844
6	17		

Estimates of seagrass coverage area (km^2) based on the voting system from Table 3, integrating estimates from current remote sensing, (18), (19), and (Schill et al. 2021).

Supplemental Data Table 3. Landsat tile acquired in 2019 and 2020 used for this study.

Path/Row	Image acquisition date
14/42	2020-12-13
14/43	
13/42	2019-01-18
13/43	
14/44	2020-07-31
12/43	2019-04-01
12/44	

Supplementary Table 4. Field data used for training OLI imagery and validation of the classifier.

Substrate	Total	Training	Validation
Sg-I	176	88	88
Sg-II	625	313	312
Sg-III	682	341	341
Sg-IV	249	125	124
NSg	331	166	165
Sand	479	240	239
Total	2542	1273	1269

Categories are broken down into the following categories: Sg-I, <25% seagrass; Sg-II, <50% seagrass; Sg-III, <75% seagrass, Sg-IV, <100% seagrass; NSg, non-seagrass; Sand, sand substrate.

Supplementary Table 5. Accuracy obtained for four seagrass density classes and two other classes using Landsat 8 OLI thematic maps, and direct ground-truth data.

	Sg-I	Sg-II	Sg-III	Sg-IV	NSg	Sand	Row total	Producer accuracy (%)
Sg-I	54	10	16	11	7	38	136	56
Sg-II	7	231	17	3	6	20	284	74
Sg-III	8	32	263	18	10	11	342	77
Sg-IV	1	19	28	81	19	11	159	65
NSg	7	15	13	11	117	4	167	71
Sand	20	5	4	1	6	145	181	63
Column total	97	312	341	125	165	229	1269	
User accuracy (%)	40	81	77	51	70	80		
Overall accuracy (%)	70.2							
Kappa coefficient	0.632							

Sg-I, II, III and IV represent seagrass cover class with <25%, <50%, <75%, and <100%, respectively; NSg represents non-seagrass substrate.

Supplementary Table 6. Seagrass cover area estimated from neural-network generated classification maps for The Bahamas.

Substrate	Area (km2)	Proportion of total area (%)
Sg-I	3954	4.5
Sg-II	5068	5.8
Sg-III	5914	6.7
Sg-IV	9770	11.1
NSg	37712	42.9
Sand	25578	29.1
Grand total	87996	

Sg-I, II, III and IV represent seagrass cover class with <25%, <50%, <75%, and <100%, respectively; NSg represents non-seagrass substrate.
