# Supplementary material for: Tiger sharks support the characterization of the world’s largest seagrass ecosystem
Source: Nat Commun. 2022 Nov 1;13:6328. doi: 10.1038/s41467-022-33926-1 (PMC9626626; doi:10.1038/s41467-022-33926-1)
Supplement: Supplementary file 2 — Description of Additional Supplementary Files [file 41467_2022_33926_MOESM2_ESM.pdf]

Title: Supplemental Movie 1. Video evidence of tiger shark habitat use of seagrass habitat.

Description: Example of video footage obtained from free-ranging, camera-mounted tiger shark on the Little Bahama Bank, with clear evidence of seagrass composing the benthic seafloor habitat.
