# Supplementary material for: Tiger sharks support the characterization of the world’s largest seagrass ecosystem
Source: Nat Commun. 2022 Nov 1;13:6328. doi: 10.1038/s41467-022-33926-1 (PMC9626626; doi:10.1038/s41467-022-33926-1)
Supplement: Supplementary file 4 — Reporting Summary [file 41467_2022_33926_MOESM4_ESM.pdf]

nature portfolio

Corresponding author(s): Gallagher AJ

Last updated by author(s): Sep 27, 2022

Reporting Summary

Nature Portfolio wishes to improve the reproducibility of the work that we publish. This form provides structure for consistency and transparency in reporting. For further information on Nature Portfolio policies, see our Editorial Policies and the Editorial Policy Checklist.

Statistics

For all statistical analyses, confirm that the following items are present in the figure legend, table legend, main text, or Methods section.

n/a	Confirmed	
☐	☒	The exact sample size (*n*) for each experimental group/condition, given as a discrete number and unit of measurement
☐	☒	A statement on whether measurements were taken from distinct samples or whether the same sample was measured repeatedly
☐	☒	The statistical test(s) used AND whether they are one- or two-sided<br>*Only common tests should be described solely by name; describe more complex techniques in the Methods section.*
☐	☒	A description of all covariates tested
☐	☒	A description of any assumptions or corrections, such as tests of normality and adjustment for multiple comparisons
☐	☒	A full description of the statistical parameters including central tendency (e.g. means) or other basic estimates (e.g. regression coefficient) AND variation (e.g. standard deviation) or associated estimates of uncertainty (e.g. confidence intervals)
☐	☒	For null hypothesis testing, the test statistic (e.g. *F*, *t*, *r*) with confidence intervals, effect sizes, degrees of freedom and *P* value noted<br>*Give P values as exact values whenever suitable.*
☒	☐	For Bayesian analysis, information on the choice of priors and Markov chain Monte Carlo settings
☒	☐	For hierarchical and complex designs, identification of the appropriate level for tests and full reporting of outcomes
☒	☐	Estimates of effect sizes (e.g. Cohen's *d*, Pearson's *r*), indicating how they were calculated

Our web collection on statistics for biologists contains articles on many of the points above.

Software and code

Policy information about availability of computer code

Data collection	Ortho-rectified Landsat 8 OLI data were downloaded from the United States Geological Survey (USGS) (https://earthexplorer.usgs.gov). The rest of the data sources (tiger shark camera and track data, seagrass data) were collected without software.
Data analysis	All code was custom and was generated and analyzed alongside data in RStudio Version 1.2.5019 and R version 4.1.0 (2021-05-18). ImageJ (v. 1.53e) is free to download from https://imagej.nih.gov/ij/download.html; QGIS (v. 3.18) is free to download from https://qgis.org/en/site/forusers/download.html; Fmask (v. 4.3) is free to download from https://github.com/GERSL/Fmask.

For manuscripts utilizing custom algorithms or software that are central to the research but not yet described in published literature, software must be made available to editors and reviewers. We strongly encourage code deposition in a community repository (e.g. GitHub). See the Nature Portfolio guidelines for submitting code & software for further information.

Data

Policy information about availability of data

All manuscripts must include a data availability statement. This statement should provide the following information, where applicable:

- Accession codes, unique identifiers, or web links for publicly available datasets
- A description of any restrictions on data availability
- For clinical datasets or third party data, please ensure that the statement adheres to our policy

All empirical data and derived estimates used in the analyses are accessible through DRYAD51. Source data are provided with this paper. The tiger shark tracking data and camera tag results from this study have been deposited in the DRYAD51 data repository. The seagrass mapping data, pro-cessed seagrass estimates and

ground-truth seafloor photogrammetry information from this study are available DRYAD51 data repository. The Allen Coral Atlas mapping data were downloaded from: https://allencoralatlas.org/atlas/#5.39/24.3807/-76.0918. Source data are provided with this paper. The processed seagrass carbon stock data and tiger shark depth data are provided in the Source Data file.

Human research participants

Policy information about studies involving human research participants and Sex and Gender in Research.

Reporting on sex and gender	n/a
Population characteristics	n/a
Recruitment	n/a
Ethics oversight	n/a

Note that full information on the approval of the study protocol must also be provided in the manuscript.

Field-specific reporting

Please select the one below that is the best fit for your research. If you are not sure, read the appropriate sections before making your selection.

☐ Life sciences ☐ Behavioural & social sciences ☒ Ecological, evolutionary & environmental sciences

For a reference copy of the document with all sections, see nature.com/documents/nr-reporting-summary-flat.pdf

Ecological, evolutionary & environmental sciences study design

All studies must disclose on these points even when the disclosure is negative.

Study description	This study describes the spatial extent of seagrass habitat in The Bahamas, using an innovative integration of data streams. These data streams included remote sensing, seafloor photogrammetry, tiger shark movements, tiger shark camera deployments, and seagrass sediment cores. No experiments were performed in this study.
Research sample	Free-ranging, wild tiger sharks (Galeocerdo cuvier) were safely tagged and released from 2011 -2019, for all shark-based data portions of the study. The nature of finding and sampling these large predators makes data collection a largely opportunistic endeavor. We combined all available data from this long-term monitoring for our study. The tiger sharks evaluated in this study were believed to be representative of the population of tiger sharks in The Bahamas, given broad geographic distribution of individuals which provided data across the two primary carbonate banks in The Bahamas.
Sampling strategy	Sample sizes were chosen based on rough comparisons to relevant, published studies for each data stream (tracking and camera tag data on large sharks such as tiger sharks, ground-truthing used in remote sensing studies, seagrass sediment cores).
Data collection	Data were collected by authors listed in the submitted co-author list, according to the Author Contributions. Field data were collected as such: Tiger shark satellite tag (AJG, ONS, NH, BDS, SK, NLP, LH), tiger shark camera tag data (AJG, BDS, SK, ABC, NLP, LH), benthic photos (AJG, BDS, AP, ASK, AM), remote sensing and mapping (JWB, MSH), and seagrass sediment core collection and analysis (AJG, WH, SDH, CF, CMD) . All data were collected either from boats during scheduled research trips in The Bahamas.
Timing and spatial scale	Data collection for the study began in January 2011 and concluded on December 16, 2020. The majority of shark data were collected from 2018-2020. All diver data were collected from 2018-2020, and remote sensing estimates were performed from May 2020 - May 2021. Seagrass sediment core samples were collected in 2011 and 2022. All empirical data were collected within the Exclusive Economic Zone of The Commonwealth of The Bahamas.
Data exclusions	No data were excluded.
Reproducibility	No attempts were made to reproduce the findings here; however, all spatial estimates were ground-truthed using real images from the Bahama Banks. Furthermore, remote sensing machine learning algorithms were validated using real field data, producing accuracy estimates as seen in the Extended Data and Supplementary Information. All aspects of the field work were performed independently (tiger shark tagging, remote sensing, seafloor photogrammetry, carbon stock analyses).
Randomization	All study organisms used were free-ranging, wild, and thus randomly sampled. All individuals from which data were collected were ultimately released alive. No experimentation was performed which may have required random sampling.
Blinding	Blinding was not relevant to this study as this was not involving human subjects nor species that were tested under any controlled experimental conditions.

Did the study involve field work? ☒ Yes ☐ No

Field work, collection and transport

Field conditions	Field data were collected from boats and by underwater divers on The Litte Bahama Bank and The Great Bahama Bank. Sea conditions ranged from 0-2 meters of surface waves, and wind varied from 0 - 35 knots.
Location	All sampling occurred within the Exclusive Economic Zone of the territorial waters of The Commonwealth of The Bahamas. Field data collection occurred, broadly, within these locations: Surveyed areas included: southern New Providence (24.948862°, -77.387834°), southeast of New Providence (24.980265°, -77.229168°), south of Rose Island (25.066268°, -77.160063°), the middle Great Bahama Bank (24.735355°, -77.212998°), and the northern Exumas (24.729973°, -76.889488°). All shark and diver data were collected in water depths from 2-25 meters.
Access & import/export	All field research was conducted within The Commonwealth of The Bahamas, approved across multiple years of consecutive permits provided from the Department of Marine Resources, Ministry of Agriculture and Marine Resources, and The Department of Environmental Planning and Protection, The Government of The Bahamas, issued to the following authors: AJG, ASK, NH. Export of seagrass sediment cores were approved via permit from The Department of Environmental Planning and Protection to AJG (no CITES permits were needed for this).
Disturbance	No disturbance was caused by this study.

Reporting for specific materials, systems and methods

We require information from authors about some types of materials, experimental systems and methods used in many studies. Here, indicate whether each material, system or method listed is relevant to your study. If you are not sure if a list item applies to your research, read the appropriate section before selecting a response.

Materials & experimental systems

n/a	Involved in the study	
☒	☐	Antibodies
☒	☐	Eukaryotic cell lines
☒	☐	Palaeontology and archaeology
☒	☐	Animals and other organisms
☒	☐	Clinical data
☒	☐	Dual use research of concern

Methods

n/a	Involved in the study	
☒	☐	ChIP-seq
☒	☐	Flow cytometry
☒	☐	MRI-based neuroimaging
